# Dengue illness impacts daily human mobility patterns in Iquitos, Peru

**DOI:** 10.1371/journal.pntd.0007756

**Published:** 2019-09-23

**Authors:** Kathryn L. Schaber, Valerie A. Paz-Soldan, Amy C. Morrison, William H. D. Elson, Alan L. Rothman, Christopher N. Mores, Helvio Astete-Vega, Thomas W. Scott, Lance A. Waller, Uriel Kitron, John P. Elder, Christopher M. Barker, T. Alex Perkins, Gonzalo M. Vazquez-Prokopec

**Affiliations:** 1 Program of Population Biology, Ecology and Evolution, Emory University, Atlanta, Georgia, United States of America; 2 Department of Global Community Health and Behavioral Sciences, Tulane School of Public Health and Tropical Medicine, New Orleans, Louisiana, United States of America; 3 Department of Entomology and Nematology, University of California Davis, Davis, California, United States of America; 4 Institute for Immunology and Informatics and Department of Cell and Molecular Biology, University of Rhode Island, Providence, Rhode Island, United States of America; 5 Department of Virology and Emerging Infections, U.S. Naval Medical Research Unit No. 6, Lima and Iquitos, Peru; 6 Department of Biostatistics and Bioinformatics, Rollins School of Public Health, Emory University, Atlanta, Georgia, United States of America; 7 Department of Environmental Sciences, Emory University, Atlanta, Georgia, United States of America; 8 Graduate School of Public Health, San Diego State University, San Diego, California, United States of America; 9 Department of Pathology, Microbiology, and Immunology, School of Veterinary Medicine, University of California, Davis, California, United States of America; 10 Department of Biological Sciences and Eck Institute for Global Health, University of Notre Dame, Notre Dame, Indiana, United States of America; Centers for Disease Control and Prevention, Puerto Rico, UNITED STATES

## Abstract

**Background:**

Human mobility plays a central role in shaping pathogen transmission by generating spatial and/or individual variability in potential pathogen-transmitting contacts. Recent research has shown that symptomatic infection can influence human mobility and pathogen transmission dynamics. Better understanding the complex relationship between symptom severity, infectiousness, and human mobility requires quantification of movement patterns throughout infectiousness. For dengue virus (DENV), human infectiousness peaks 0–2 days after symptom onset, making it paramount to understand human movement patterns from the beginning of illness.

**Methodology and principal findings:**

Through community-based febrile surveillance and RT-PCR assays, we identified a cohort of DENV+ residents of the city of Iquitos, Peru (n = 63). Using retrospective interviews, we measured the movements of these individuals when healthy and during each day of symptomatic illness. The most dramatic changes in mobility occurred during the first three days after symptom onset; individuals visited significantly fewer locations (Wilcoxon test, p = 0.017) and spent significantly more time at home (Wilcoxon test, p = 0.005), compared to when healthy. By 7–9 days after symptom onset, mobility measures had returned to healthy levels. Throughout an individual’s symptomatic period, the day of illness and their subjective sense of well-being were the most significant predictors for the number of locations and houses they visited.

**Conclusions/Significance:**

Our study is one of the first to collect and analyze human mobility data at a daily scale during symptomatic infection. Accounting for the observed changes in human mobility throughout illness will improve understanding of the impact of disease on DENV transmission dynamics and the interpretation of public health-based surveillance data.

## Author summary

Dengue is the most important mosquito-borne viral disease of humans worldwide. Due to the limited mobility of the mosquitoes that transmit dengue virus, human mobility can be a key to both understanding an individual’s exposure to the virus and explaining the spread of dengue throughout a population. Accurate disease models should include human mobility; however, changes in human movement patterns due to the presence of symptoms need to be taken into account. We quantified the impact of symptom presence on human mobility throughout the infectious period by analyzing a dataset on the daily movements of dengue virus infected individuals. Accounting for these changing patterns of mobility will improve understanding of the complex relationship between symptom severity, human movement, and dengue virus transmission. Furthermore, dengue transmission models that incorporate symptom-driven mobility changes can be used to evaluate scenarios and strategies for disease prevention.

## Introduction

Human mobility plays a central role in shaping the structure of transmission networks and in influencing epidemiologic processes such as pathogen introduction, epidemic transmission, and endemic persistence [1–4]. While human mobility can drive transmission across multiple spatial and temporal scales [3, 5], it is at the finest scales (daily, intra-urban human movements) where epidemic processes occur and emergency public health interventions are usually implemented. Evidence from theoretical models and empirical studies show that individual and/or spatial variability in number and frequency of contacts can lead to transmission heterogeneity, where certain individuals or locations contribute disproportionately to pathogen transmission and epidemic spread [6–8]. Thus, identifying social and behavioral characteristics (e.g., mobility patterns, occupations, age classes) most responsible for such disproportionate contributions has become a public health priority, with significant potential for leveraging the power of public health surveillance programs and targeted disease control [9–11].

Dengue, an acute illness caused by four immunologically related viruses in the family *Flaviviridae* and transmitted by *Aedes* spp. mosquitoes (primarily *Aedes aegypti*), is the most important mosquito-borne viral disease of humans worldwide [12]. Because *Aedes aegypti* seldom disperse beyond 100 meters, have a propensity for resting and biting inside residential buildings, and bite during the day [13–16], human movements are key to explaining the urban transmission dynamics of dengue virus (DENV) [17–21]. Individual movement patterns can also expand the spatial scale of transmission and lead to significant heterogeneity in transmission patterns by connecting otherwise discrete subpopulations of mosquitoes [3, 22, 23]. Extensive movement studies performed in the upper Amazon city of Iquitos, Peru, have shown that while human mobility within a resource-poor urban center is highly unstructured (with only 38% of participants having regular mobility routines), the majority of locations visited are either residential or commercial, with most movements (81%) occurring within 1 km of an individual’s home [24–28]. Moreover, an individual’s risk of DENV infection significantly increased when he or she routinely visited the same residential locations as DENV-infected people, whereas the distance the individual lived from a DENV-infected case was not significant [26].

Such empirical characterizations of fine-scale human mobility patterns and risk of DENV infection have informed complex simulation models that explore the theoretical role of human movement on the spatial and temporal patterns of disease dynamics [18, 23, 29]. When mobility-driven contact structure is included in theoretical models, the effect on DENV epidemic transmission is dramatic. Overlapping movement patterns within social groups drive the fine-scale heterogeneity in DENV transmission rates; however, the presence of a mosquito vector can hide the effect of socially structured movements if only spatially aggregated infection dynamics are considered [23]. Such models do not take into account the fact that symptomatic infection may influence mobility, which in turn can influence onward virus transmission and the structure of transmission chains. Research on directly transmitted diseases has demonstrated disease-driven behavior changes [30] and the significant influence they can have on predictions of pathogen spread [31–34]. For DENV, mobility data have been captured for febrile symptomatic individuals and healthy individuals using either retrospective movement surveys [35] or GPS trackers [36]. Febrile DENV-infected individuals visited significantly fewer places, traveled shorter distances, and spent more time at home [35, 36]. These patterns reveal particularly important information for understanding the complex relationship between symptom severity and human mobility, and to ultimately determine if there is an association between human mobility and infectiousness to mosquitoes. However, because DENV infectiousness peaks at 0–2 days after onset of symptoms and lasts for 4–5 days after onset of symptoms [37–39], human mobility during the first few days of symptoms could be key to better understand transmission dynamics. The goal of this study, therefore, was to conduct detailed, daily retrospective interviews to measure the mobility behavior of clinically apparent DENV-infected individuals throughout their illness, with the goal of generating mobility metrics that can be used to characterize the absolute and relative impacts of disease on potential exposure to *Aedes aegypti* mosquitoes.

## Methods

### Study area

This study was performed in the Amazon city of Iquitos, Peru. Iquitos is a geographically isolated, tropical urban environment with approximately 430,000 inhabitants located along the margin of the Amazon River [40]. The city’s economic structure is highly informal and dynamic, with one-third of economically active individuals either unemployed or informally employed [41]. Iquitos has been the home of extensive, long-term arboviral research led by the University of California, Davis and U.S. Naval Medical Research Unit 6 since 1999 [3, 24–28, 42]. Extensive human mobility studies paired with detailed epidemiological data have made Iquitos an informative site for understanding the dynamics of arbovirus transmission. All four serotypes of DENV have been introduced in Iquitos; however, at any particular time virus transmission is usually dominated by a single serotype [42, 43]. Previous research [27] demonstrated that inhabitants visit an average of 5.8 (±3.6 SD) locations over a two-week period. While most movement (~80%) occurs within 1 km of their home, inhabitants have highly irregular and temporally unstructured routines that are not dominated by a single location, such as a workplace [27].

### Study design

The study followed a contact-cluster design in which reverse transcription polymerase chain reaction (RT-PCR) positive, or viral nucleic acid test positive, DENV-infected individuals (index cases) were captured through community or clinic-based febrile surveillance systems, as described previously [26]. At the time of the initial blood sample, a 15-day retrospective semi-structured movement survey (RMS) was administered to the index case to identify the locations they visited in the 15 days prior to diagnosis (characterizing the “pre-illness” period). Consenting individuals (contacts) from the index cases’ home and residential locations visited by the index case were then screened for DENV infection using RT-PCR [26] [44]. The RMS was administered to DENV PCR-positive contacts to quantify mobility behavior associated with potential virus exposure.

RMSs were developed based on findings of focus groups and validated by comparison with data from people wearing GPS tracking devices [24, 25]. RMSs capture positional, temporal and behavioral information of routine human mobility. Questions focus on the amount of time an individual spent at home, the visitors they received, and the places they visited. For time spent at home, individuals were asked about the average number of hours spent at home each day of the week, specifically focusing on the period from 5 a.m. to 10 p.m., which includes the peak landing and biting times for *Aedes aegypti* [45]. For places visited, information was collected on the type of place visited, when, for how long, and how often in the 15-day period. Trained, local Iquitos residents (the ‘Movement Team’) verbally administered electronic RMSs and recorded the information on tablets in the CommCare application [46].

To track movements of DENV positive participants during their illness, daily interviews using a modified daily RMS (DRMS) were conducted in person or by telephone for 7 days following the initial RT-PCR-positive blood test (S1 File). Where participants were not available for daily interviews, information about movements on several days was collected at a single interview. The DRMS asks about the amount of time spent at home the previous day(s) and the following information about each place visited during the previous day(s): day visited, place type, location, time of day visited, and time spent. For residential places visited, the DRMS asks whom they were visiting, their reason for visiting, if anyone in the home was ill during the preceding 15 days, and (for routinely visited houses) if/why there was any change in the time of visitation, as compared to the “pre-illness” period. During this seven-day period, DENV positive individuals were also administered two Quality of Well-Being surveys (QWB) by the Movement Team, one 2–3 days and one 7 days after the initial PCR-positive blood test. The QWB survey is a validated instrument used to measure an individual’s quality of life during chronic illness [47]. Our study was a novel application of the QWB survey to an acute illness. The survey responses were sent to the developers at University of California, San Diego, who used a weighted algorithm to produce one well-being score between 0.0 (death) and 1.0 (asymptomatic and fully-functioning) covering the three days prior to each survey date [47].

At a follow-up visit scheduled 30 days after the initial PCR-positive blood test, individuals were given a 15-day (“post-illness”) RMS and QWB survey in an effort to record their “baseline” mobility behavior and well-being in the absence of illness.

### Data processing

For each study participant, the following variables were computed from the “pre-illness” and “post-illness” 15-day RMS: (1) total number of locations visited, (2) proportion of visits to each location type, (3) total number of houses visited, (4) proportion of visits to houses of family members vs. houses of friends, and (5) average proportion of time spent at home per day. Equivalent daily values of these variables were collected for each participant from the DRMSs. Rather than referring to values as occurring on a certain number of days after the PCR-positive blood test, a standardized “day after symptom onset” variable was calculated. Because blood tests were not done on the same day of illness for all participants, DRMSs captured a range of 1–15 days after symptom onset. We focused our analysis on days 1–9 after symptom onset; few individuals had data for days 10–15 after symptom onset.

### Data analysis

Analysis of mobility data had two main objectives: (1) comparing healthy (pre- and post- illness) mobility to mobility during illness, and (2) determining if mobility patterns changed during the 9 days after symptom onset.

For the first objective, mobility during illness was calculated by averaging a participant’s DRMS for all available time points up to day 9 after symptom onset. Comparisons were done for the following mobility metrics: daily number of locations visited, daily number of houses visited, and proportion of time spent at home. When a metric followed a normal distribution (assessed via the Shapiro-Wilk test), pairwise comparisons were performed with paired t-tests followed by Holm-Bonferroni corrections. When the variable was not normally distributed, the non-parametric Kruskall-Wallis Rank Sum Test and pairwise Wilcoxon Signed Rank Test for paired data were utilized. As many individuals would stop visiting other locations during their illness period [35], we also analyzed the number of locations, number of houses, and time at home as binary variables, asking if any locations/houses were visited and if any time was spent away from home. These binary outcomes were compared between all possible pairs of time points (pre-during, during-post, and pre-post illness) using McNemar’s χ^2^ test.

If locations were visited, further analyses determined what type of locations they were. While these data were subject-correlated across time points, they could not be analyzed as paired data because not all participants visited locations at every time point. Generalized logistic mixed-effects models (GLMMs) determined the association between the probability of a location type being visited and the time period being considered (pre-during-post illness), while accounting for repeated measures by using participant ID as the random intercept. Location type was separated into four groups: (1) house, (2) health, (3) education/work, and (4) other (e.g., recreation, church, market, port). Similarly, logistic GLMMs determined the association between time period (pre-during-post symptoms) and the probability of a specific house type being visited (e.g., family versus friend).

For the second objective, aiming to determine whether mobility patterns changed during the illness period, we calculated mobility metrics for 3-day groups (days 1–3, 4–6, and 7–9 after onset of symptoms). Daily data were aggregated into 3-day groups to allow for robust analyses, while also controlling for the dearth of data points on certain days. In particular, the first two days after symptom onset had incomplete information for some participants due to the time required to capture individuals with symptoms, run RT-PCR tests, and obtain confirmed test results. To make pre/post and during-illness data comparable, 15-day RMS values were condensed to give movements over an average 3-day period. Analysis of the number of locations/houses, proportion of location/house types, and time spent at home followed the same steps described above. Comparisons were made between the 3-day groups to determine whether significant changes occur in movement patterns during illness. Further, movements within the 3-day groups were each compared to post-illness mobility.

The associations between daily (DRMS) mobility patterns and possible predictor variables were examined using Generalized Linear Mixed Models (GLMMs), Generalized Additive Mixed Models (GAMMs), and Generalized Additive Models for Location Scale and Shape (GAMLSSs) [48–50]. Best-fit models were determined for each of the following mobility outcomes: total number of locations visited (count variable), relative number of locations visited (compared to when healthy) (continuous variable), total number of houses visited (count), relative number of houses visited (continuous), total proportion of time spent time at home (continuous), and relative amount of time spent at home (continuous). For both the total number of locations visited and total number of houses visited, GLMMs and GAMMs with underlying Poisson distributions were compared. An individual’s age, occupation, gender, QWB score, and the “day after symptom onset” were considered as predictor variables, with the best-fit model determined using a AIC and a Chi-square test comparing reduction in residual deviance. The response variable proportion of time spent at home was best characterized by a one-inflated beta distribution, so analysis was done with GAMLSS, as detailed below.

Although GLMM and GAMM regressions model the mean (μ) value of the distribution of the response variable, GAMLSS allows other distribution parameters to be modeled as a function of explanatory variables. A one-inflated beta distribution has possible values 0<y≤1 and is defined in two parts: the probability that y = 1 (modeled by the η parameter) and the probability for 0<y<1, which is shaped by a traditional beta distribution with parameters mean (μ) and shape (σ). Here, the η parameter was the probability an individual stayed at home 100% of the time (y = 1). If an individual did not stay at home the entire day, the proportion of time that was spent at home (0<y<1) was determined by a beta distribution with μ and σ.

We also considered response variables as relative values in order to control for the individual variation in mobility levels. The number of locations (houses) an individual visited on each day during illness was considered relative to the average number of locations (houses) they visited pre-illness. Similarly, the number of hours a participant spent at home during each day of illness was compared to the average number of hours that individual spent at home pre-illness. While relative number of houses could not be well explained by a set distribution, both relative number of locations visited and relative amount of time spent at home were best characterized by the logistic distribution. Analysis of these response variables was done with GAMLSS, where both the mean (μ) and the standard deviation (σ) parameters of the logistic distribution could be modeled as a function of explanatory variables. Best-fit GAMLSS models were chosen using forward and backward selection for each of the explanatory variables. All statistical analyses were performed in R 3.3.0 statistical computing software [48–51].

### Ethics statement

The procedures for enrollment of participants, dengue diagnosis, semi-structured interviews, and participant follow-up were approved by the Institutional Review Board (IRB) of the United States Naval Medical Research Center Unit No. 6 (NAMRU-6) (NAMRU6.2014.0021) in compliance with all applicable federal regulations governing the protection of human subjects. IRB relying agreements were established between NAMRU-6 and Emory University, Tulane University, University of California Davis, University of Rhode Island, San Diego State University, and University of Notre Dame. In addition to IRB approval, investigators obtained host country approval from the Loreto Regional Health Department, which oversees health research in Iquitos. Adult study participants provided written informed consent and a parent or guardian provided informed consent on behalf of child study participants.

## Results

Detailed mobility data were collected from a total of 62 DENV+ participants. Descriptions of participant demographics and data completeness appear in the Supplemental Text. The most commonly reported symptoms were general malaise (100%), weakness (96.61%), fever (93.22%), headache (91.53%), anorexia (89.83%), and musculoskeletal pain (84.75%). During days 1–9 post-onset of symptoms, the average maximum malaise intensity of participants was 7.5 on a scale of 10 (range: 0–10), as compared to a mean intensity of 0.28 out of 10 (range: 0–6) during the post-illness time period. While all participants reported some level of malaise and dengue-related symptoms, the vast majority of participants (88.7%) received only outpatient care.

For the 34 participants with QWB scores collected at all time points (2–3 days post blood test, a week post blood test, and post-illness), scores were considered in terms of “days after symptom onset”. The mean QWB score of those reported in the nine days after symptom onset was 0.61 (range: 0.25–1.0). The median QWB score was less than 0.70 all nine days after symptom onset; however, a few individuals had scores of 1.0 (“asymptomatic”) as early as day 4. While the DRMS may have captured mobility on days when individuals were asymptomatic, the vast majority of individuals retained symptoms throughout the nine days after symptom onset (S3 Fig). Therefore, that period will be referred to as “during illness” in the remaining sections.

The mean QWB score at the post-illness time point was 0.88 (range: 0.48–1.0, median 1.0). While the post-illness survey may contain data from a few individuals who were still symptomatic, in general we considered it a suitable approximation of healthy mobility. Another proxy for healthy mobility, the “pre-illness” RMS, may have also captured some symptomatic movements because the 15-day retrospective survey could not be given until DENV cases were captured a median of 3 days after symptom onset.

### Healthy vs. symptomatic mobility patterns

**Fig 1:**
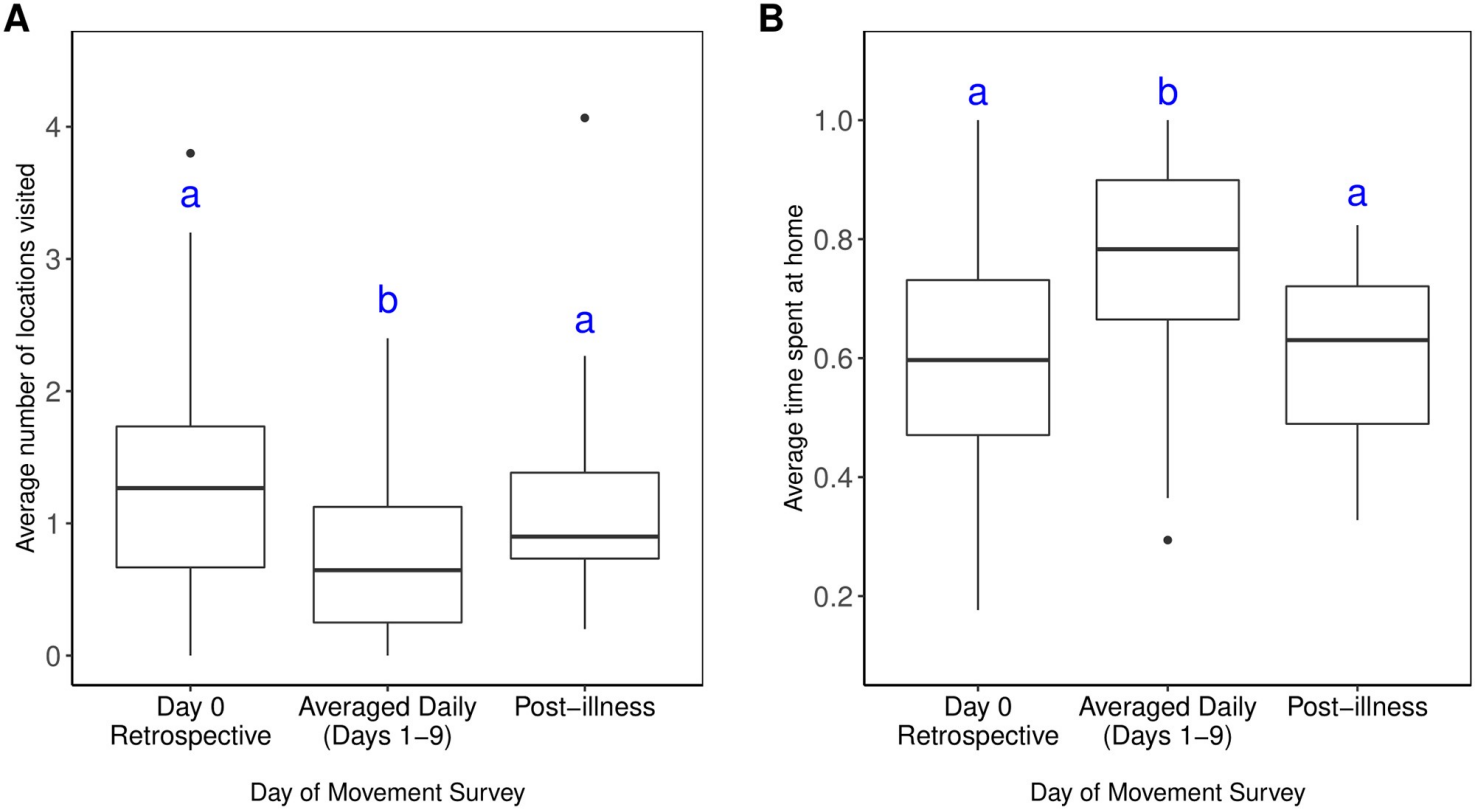
Mobility values throughout illness. (A) Average number of locations visited during each time period (B) Average proportion of time spent at home during each time period. Significant differences, denoted by letters, were detected using pairwise paired Wilcoxon Sign Rank tests with Bonferroni’s correction to account for a family-wise error-rate of 0.05. All significant differences had p-values < 0.01.

When comparing healthy (pre-/post-illness) and symptomatic time points, there was a significant difference in both the proportion of time spent at home and the average number of locations visited (Fig 1A and 1B). Healthy participants spent 60% of their time at home and visited an average of 1.3/1.1 (pre-/post-illness) locations per day, whereas ill participants spent 74% of their time at home (Wilcoxon test: p < 0.001) and visited an average of 0.73 locations (Wilcoxon test: pre-illness: p < 0.001; post-illness: p = 0.010) (S2 Table). Participants were also significantly less likely to visit other houses during illness, as compared to pre-illness (McNemar’s χ^2^: p < 0.001) and post-illness (McNemar’s χ^2^: p = 0.043) (S3 Table).

**Fig 2:**
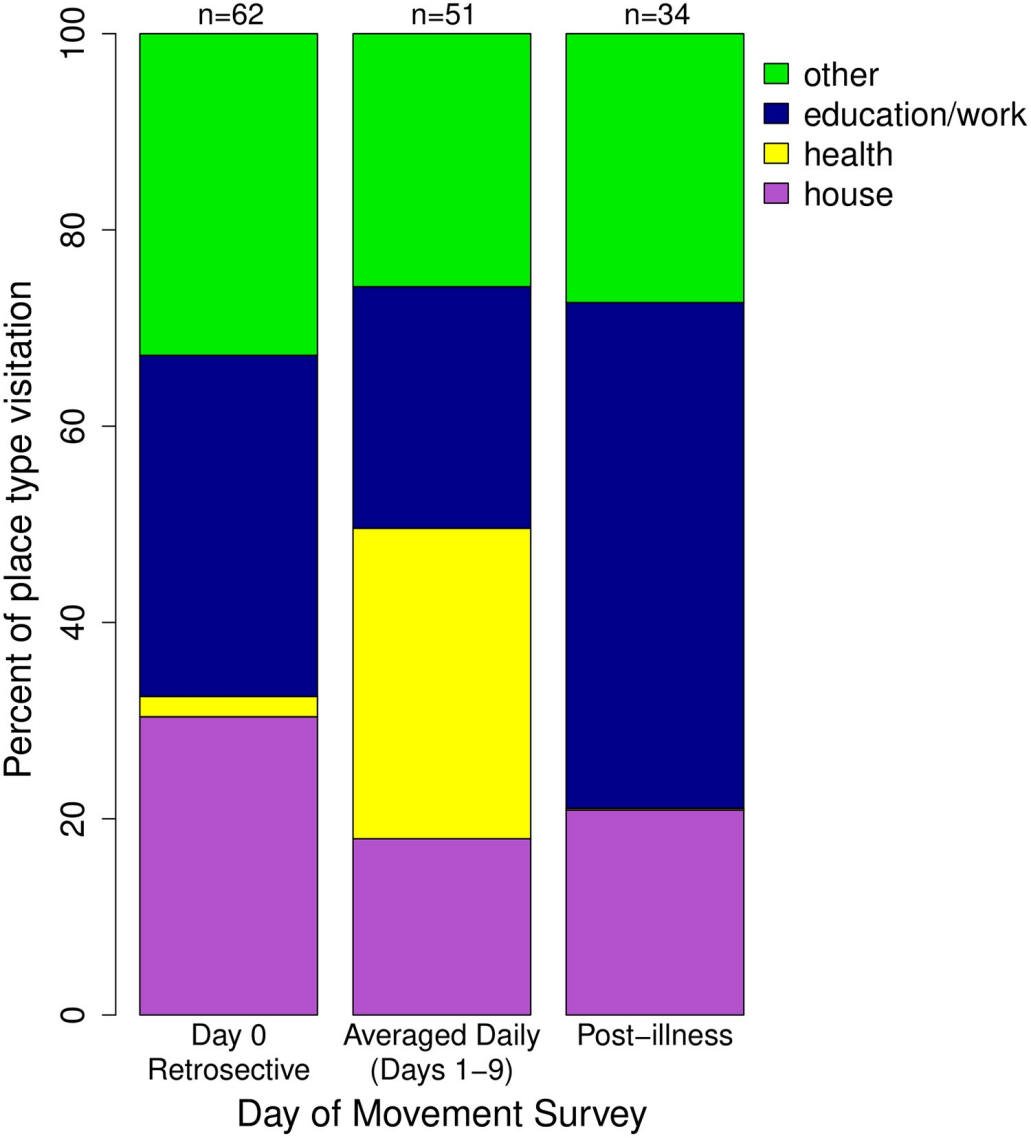
Place types visited throughout illness. Expressed as the percent of locations being visited of each location type. Other location type includes: markets, restaurants, ports, churches, cemeteries, recreational places, internet cafes, and all else. The number of participants who visited places is listed above each time point.

The odds (adjusted odds ratio/AOR) of an individual visiting an education/work location during healthy time points (AOR pre-/post-illness: 2.0/4.4) were significantly greater than during illness (GLMM: p < 0.001; Table 1, Fig 2). Similar significant differences were seen for visits to “other” place types (GLMM: p<0.001; Table 1, Fig 2). Conversely, the odds of participants going to a health-related place pre- or post-illness were significantly lower than during illness (AOR: pre-/post-illness: 0.019/0.002; Table 1, Fig 2). Although individuals were more likely to visit a house during the pre-illness time period as compared to during illness (AOR: 1.684; GLMM: p = 0.013), there was no significant difference for post-illness (AOR: 0.872; GLMM: p = 0.64), where individuals were predicted to visit houses with a mean probability of 21% (Table 1, S4 Table).

**Table 1:** Adjusted odds ratios (and 95% Confidence Intervals) for location type throughout illness, derived from logistic GLMMs.

	Pre-Illness (Day 0)	Post-Illness (Day 30)
**Education/Work**	1.884 (CI: 1.317–2.725) ***	4.429 (CI: 2.964–6.695) ***
**Health**	0.019 (CI: 0.009–0.037) ***	0.002 (CI: 0.001–0.008) ***
**Other**	2.144 (CI: 1.496–3.110) ***	1.660 (CI: 1.109–2.508) *
**House**	1.684 (CI: 1.110–2.585) *	0.872 (CI: 0.539–1.419)
**Family’s House**	0.348 (CI: 0.078–1.360)	0.509 (CI: 0.125–1.855)

Odds Ratios are for pre- and post-illness time periods, compared to the period during illness (daily interview period) based on logistic GLMMs. For houses, the odds ratios are given for family member’s houses (vs. friend’s houses). Significant associations between time period and location visitation are denoted with asterisks (* p<0.05, ** p<0.01, ***p<0.001).

### Mobility patterns during illness

**Fig 3:**
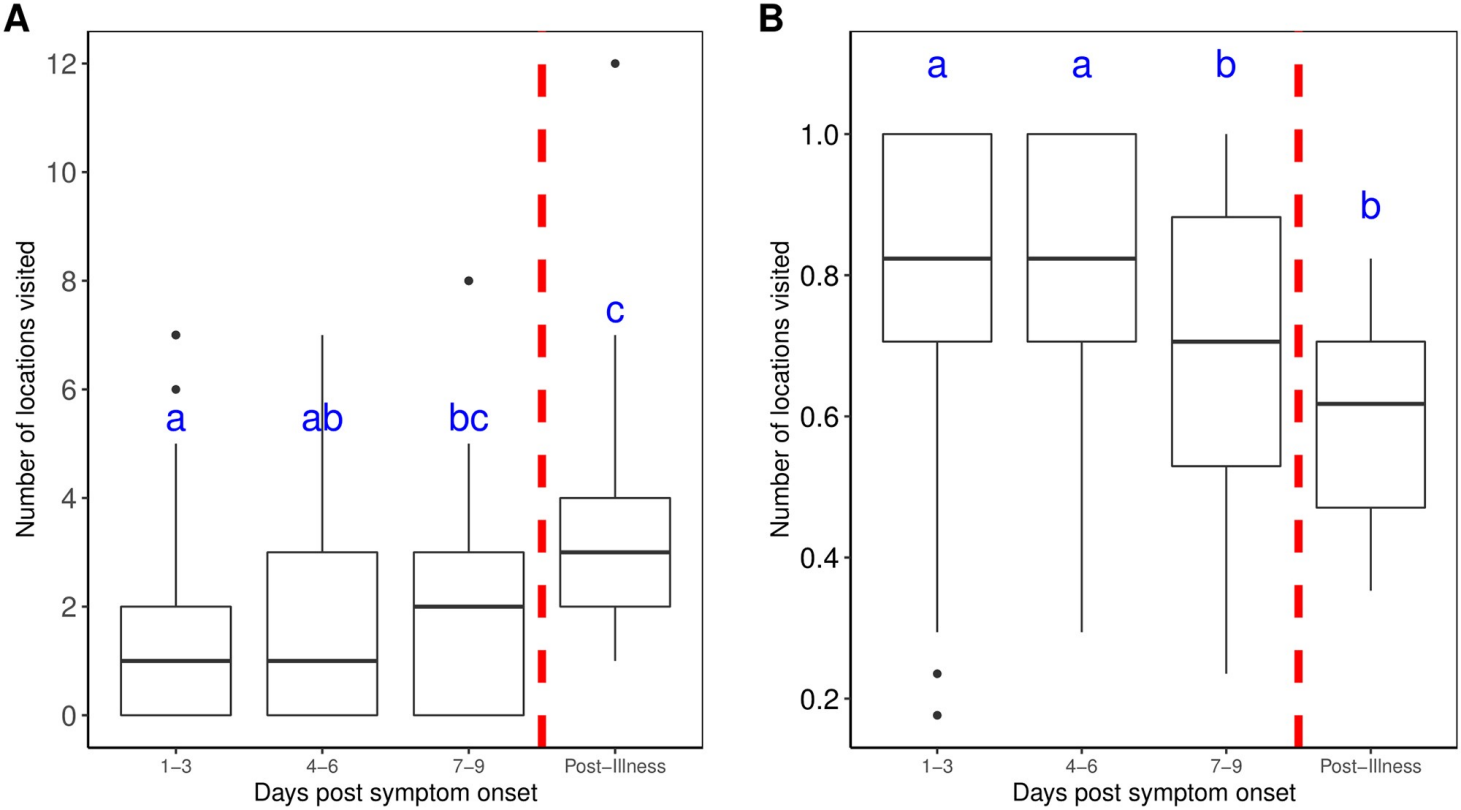
Mobility values during illness (in 3-day intervals). (A) Average number of locations visited per 3-day period. (B) Average proportion of time spent at home per 3-day period. Significant differences, denoted by letters, were detected using pairwise paired Wilcoxon Sign Rank tests with Bonferroni’s correction to account for a family-wise error-rate of 0.05. All significant differences had p-values < 0.05.

During days 1–3 and 4–6 after symptom onset, individuals were significantly more likely to spend all of their time at home, compared to both days 7–9 after symptom onset (McNemar’s χ^2^: p = 0.046) and post-illness (McNemar’s χ^2^: days 1–3: p = 0.008; days 4–6: p = 0.008) (S6 Table). There was also a significant difference in the average proportion of time spent for days 1–3 and 4–6 (76%) when compared to both days 7–9 (69%) (Wilcoxon test: days 1–3: p = 0.014; days 4–6: p = 0.008) and post-illness (59%) (Wilcoxon test: days 1–3: p = 0.005; days 4–6: p < 0.001; Fig 3B, S5 Table). Individuals were significantly less likely to visit any locations during illness compared to post-illness (McNemar’s χ^2^: days 1–3: p = 0.001; days 4–6: p < 0.001; days 7–9: p = 0.008; S6 Table). Accordingly, the average number of locations visited was significantly lower on days 1–3 (paired t-test: p = 0.017) and 4–6 after symptom onset (paired t-test: p < 0.001) when compared to the mean 3.4 places visited every 3 days at post-illness (Fig 3A). The average number of locations visited on days 1–3 (1.5 places/3-days) was also significantly less than the average number of locations visited on days 7–9 after symptom onset (2.2 places/3-days) (Wilcoxon test: p = 0.047; S5 Table).

**Fig 4:**
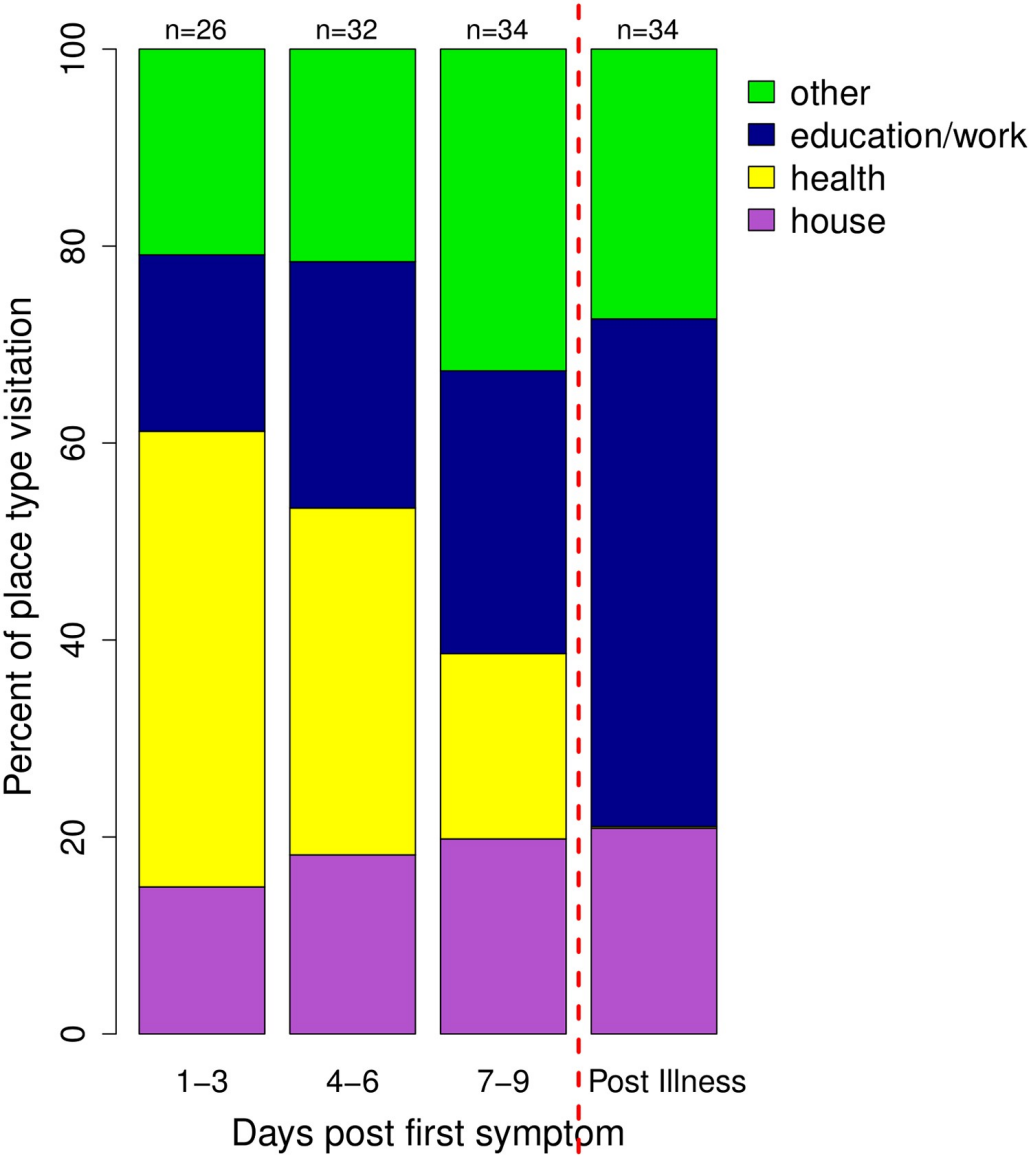
Place Types visited during illness (in 3-day intervals). Expressed as the percent of locations being visited of each location type. Other location type includes: markets, restaurants, ports, churches, cemeteries, recreational places, internet cafes, and all else. The number of participants who visited places is listed above each time point.

When considering the type of location visited (Fig 4), the three during-illness time points (days 1-3/4-6/7-9) were compared to the post-illness period. Post-illness, the participants were predicted to visit education/work places with a 48% probability, “other” places with a 32% probability, houses with a 20% probability, and health-related places with only a 0.2% probability (S7 Table). Compared to post-illness, the odds of an individual visiting an education/work place were significantly lower for days 1–3 (AOR: 0.08), days 4–6 (AOR: 0.22), and days 7–9 after symptom onset (AOR: 0.26) (GLMM: p< 0.001; Table 2). Conversely, the odds of visiting a health-related place during illness were significantly higher compared to post-illness (AOR: days 1–3: 3826; days 4–6: 1041; days 7–9: 365), likely due to the very low probability of a health-related location being visited post-illness when healthy (GLMM: p < 0.001). The likelihood of visiting a house during illness was not significantly different than the likelihood post-illness (AOR: days 1–3: 1.08; days 4–6: 1.20; days 7–9: 1.64; GLMM: p > 0.05). There were also no significant correlations between the illness time point and the odds of visiting a family member’s (versus friend’s) house (Table 2, S7 Table).

**Table 2:** Adjusted odds ratios (and 95% Confidence Intervals) for location type during illness, derived from logistic GLMMs.

	Days 1–3	Days 4–6	Days 7–9
**Education/Work**	0.08 (CI: 0.03–0.18)***	0.22 (CI: 0.11–0.43)***	0.26 (CI: 0.14–0.45)***
**Health**	3826 (CI: 585–42793)***	1041 (CI: 205–9101)***	365 (CI: 72–3175)***
**Other**	0.51 (CI: 0.22–1.08)	0.29 (CI: 0.14–0.56)***	0.86 (CI: 0.47–1.52)
**House**	1.08 (CI: 0.39–2.80)	1.20 (CI: 0.52–2.73)	1.64 (CI: 0.76–3.42)
**Family’s House**	21.15 (CI: 0.16–1.48x10^23^)	1.48 (CI: 0.22–12.35)	0.97 (CI: 0.15–6.40)

Odds ratios are for days 1–3, 4–6, and 7–9 after symptom onset, as compared to the post-illness time period, based on logistic GLMMs. For houses, the odds ratios are given for family member’s houses (vs. friend’s houses). Significant associations between time period and location visitation are denoted with asterisks (* p<0.05, ** p<0.01, ***p<0.001).

### Daily mobility patterns during symptomatic illness

The best-fitting model to describe the relative number of locations visited was a GAMLSS with a logistic distribution. The μ parameter (mean) was best explained by a positive effect of day after symptom onset (p < 0.001) and a random intercept for participants, which allowed the mean relative number of locations to vary by participant. The σ parameter (standard deviation) was best explained by QWB score (p < 0.001), day after symptom onset (p < 0.001), and an interaction between the two (p < 0.001) (Table 3). For relative amount of time spent at home, the best-fit model was a GAMLSS with underlying logistic distribution, where the μ parameter was best explained by a negative effect of day (p = 0.0011) and a random intercept for participants. The σ parameter was best explained by a positive effect of day after symptom onset (p < 0.001) (Table 4).

**Table 3:** Fixed effects of a GAMLSS predicting relative number of locations visited, as compared to pre-illness.

Parameter		Estimate	Std. Error	z value	p-value
**μ**	Intercept	-1.07	0.023	-46.79	< 0.001 ***
**μ**	Day	0.063	0.0132	4.81	< 0.001 ***
**σ**	Intercept	-7.001	0.914	14.85	< 0.001 ***
**σ**	Day	1.086	0.157	-2.85	0.001 ***
**σ**	QWB score	7.371	5.07	1.70	0.001 ***
**σ**	Day*QWB score	-1.252	-5.56	1.70	0.001 ***

Each distribution parameter (μ, σ) has distinct explanatory variables.

μ, mean of logistic distribution; σ, standard deviation of logistic distribution

* p < 0.05,

** p < 0.01,

*** p < 0.001

**Table 4:** Fixed effects of a GAMLSS predicting relative amount of time spent at home, as compared to pre-illness.

Parameter		Estimate	Std. Error	z value	p-value
**μ**	Intercept	4.974	0.385	12.91	< 0.001 ***
**μ**	Day	-0.295	0.088	-3.37	0.0011 **
**σ**	Intercept	-0.133	0.164	-0.81	0.421
**σ**	Day	0.107	0.029	3.69	< 0.001 ***

Each distribution parameter (μ, σ) has distinct explanatory variables.

μ, mean of logistic distribution; σ, standard deviation of logistic distribution

* p < 0.05,

** p < 0.01,

*** p < 0.001

With proportion of time spent at home as the response variable, the best-fit model explains the η parameter as a function of age (<18 or >18) (p = 0.005) and an interaction between age and day after symptom onset (p = 0.018). The μ parameter was explained by a random slope of participants over time and the σ parameter was explained by the QWB score and a smoothed function of the day of illness (Table 5). This suggests that whether an individual spent all (100%) of their time at home was dependent on both their age and the day of illness, whereas the proportion of time spent at home (when less than 100%) depended on the day of illness (p = 0.005) and how they were feeling (QWB score) (p = 0.094; Table 5). While the day of illness did not have an overall effect on the mean proportion of time spent at home (when less than 100%), the random slope for participants suggests that day of illness had a varying effect across participants.

**Table 5:** Fixed effects of a GAMLSS predicting proportion of time spent at home.

Parameter		Estimate	Std. Error	z value	p-value
**μ**	Intercept	0.599	1.02e-05	58426	< 0.001 ***
**σ**	Intercept	9.147	0.616	14.85	< 0.001 ***
**σ**	pb(Day)	-0.332	0.116	-2.85	0.006 **
**σ**	QWB score	1.754	1.034	1.70	0.094
**η**	Intercept	-1.995	0.930	-2.15	0.035 *
**η**	Age: (<18)	2.992	1.044	2.87	0.005 **
**η**	Day	0.215	0.154	1.40	0.166
**η**	Age: (<18)*Day	-0.425	0.176	-2.42	0.018 *

Each distribution parameter (μ, σ, η) has distinct explanatory variables.

μ, mean of beta distribution; σ, precision of beta distribution; η, parameter for probability at one

* p < 0.05,

** p < 0.01,

*** p < 0.001

## Discussion

We found that dengue illness affects almost all aspects of an individual’s mobility behavior. During mild symptomatic illness, individuals visited significantly fewer locations and houses and spent significantly more time at home. Further, symptomatic participants visited education/work and “other” locations less often than when they were healthy and visited health locations more often. These results (1) are consistent with and expand prior evidence indicating that individuals with symptomatic illness move less than healthy individuals [31, 35, 52]; (2) refine estimates of the effects of mild symptomatic dengue illness on movement by quantifying changes before, during and after the symptomatic phase of infection; and (3) suggest the need to better account for disease-driven mobility behavior changes in DENV transmission models [31, 53].

The most dramatic changes in mobility occurred during the first 3 days after symptom onset, when significantly fewer locations were visited and significantly more time was spent at home. During days 4–6 and 7–9 after symptom onset, the number of locations visited increased and the proportion of time spent at home decreased. By days 7–9 after symptom onset, the number of locations visited and the time spent at home were no longer significantly different from healthy behaviors. This reduction in mobility during illness, particularly on days 1–3 after symptom onset, could affect an individual’s contribution to onwards DENV transmission. For DENV, viremia reaches levels infectious to mosquitoes a few days prior to symptom onset and peaks at 0–2 days after symptom onset, with titers then lowering by days 4–5 (although some individuals are still capable of infecting mosquitoes) (37–39). During peak infectiousness, most individuals are spending more time at home and visiting fewer places, thereby reducing the number of distinct *Aedes aegypti* mosquitoes with whom potential virus-spreading contacts occur. This may allow those few individuals who do not alter their movements to have a more significant role in pathogen transmission during peak infectiousness. During the pre-symptomatic period, however, almost all individuals have high mobility and a viremia level sufficient for virus transmission to mosquitoes [38]. Recent theoretical models of within-host viral dynamics for symptomatic individuals estimate that 24% of onward transmission results from mosquitoes biting during the pre-symptomatic period [54]. When also accounting for mobility changes throughout viremia, many individuals may have their greatest contribution to transmission be during the pre-symptomatic stage. Ten Bosch et. al. also estimated that asymptomatic individuals had only 80% the net infectiousness of symptomatic individuals [54]. This reduction in net infectiousness may be counteracted by the hypothetically unaltered mobility patterns exhibited by asymptomatic (and minimally symptomatic) individuals, further increasing the overall contribution of silent transmission. Such potential dynamics emerging from the coupling between individual infectiousness, movement, and disease severity deserve further investigation [55], because they may help explain the explosive nature of DENV outbreaks and the limitations of vector control in containing virus transmission.

Throughout an individual’s illness period, we found that day of illness and the participant’s subjective sense of well-being (QWB score) were significant predictors for the relative number of locations visited, as compared to pre-illness. When considering the proportion of time spent at home, an individual’s age and their day of illness were significant in predicting whether they chose to stay at home 100% of the day or not, with children being more likely to stay home all day compared to adults. When an individual chose to spend some amount of time outside their house, the day of illness and the QWB score significantly predicted the proportion of time. Further, the relative amount of time participants spent at home compared to pre-illness was also significantly predicted by the day of illness. Individuals with more severe symptoms and those at the beginning of their illness were more likely to be spending more time at home (both absolute proportion of time and amount of time relative to pre-illness values). Further, when compared to pre-illness, individuals at the beginning of their illness have lower values of relatively visited locations compared to toward the end of illness.

One limitation of our study is the reliance on participant recall, which can be subject to recall bias. However, the retrospective semi-structured interview we utilized was previously tested in Iquitos and was found to obtain superior data on activity space, as compared to wearable GPS data-loggers [24]. Further, in the DRMS participants only needed to recall movements over the past 24 hours, making bias less likely. Our study also faced limitations with the number of participants and the ability to measure movement on the first two days after symptom onset. Nevertheless, our study is one of the first to collect human mobility data at a daily scale during symptomatic infection. Future studies could build on our study by collecting detailed mobility data from more individuals with a wider spectrum of symptom severity, including across a wider range of diseases. Future studies should also seek to make coupled measurements of an individual’s infectiousness throughout the course of mobility data collection.

Human mobility patterns have played an important role in recent vector-borne disease transmission models [56]. There is, however, an increasing need to include differing mobility patterns when modeling individuals that are ill versus healthy. We demonstrate that individuals with dengue spend significantly more time at home, particularly during the first days after symptom onset when they are most infectious, potentially limiting contact with *Ae*. *aegypti* outside their home. When looking at the locations being visited during illness, however, the proportion of houses was consistent throughout and remained similar to the post-illness level. This may be of particular importance for onward transmission given the propensity for *Ae*. *aegypti* to bite inside houses [13, 14, 57]. The abundance of mosquitoes in both an individual’s home and the houses/locations they visit when infectious will likely determine the effect that reduced mobility has on their overall contribution to DENV transmission. Reduction in mobility patterns when symptomatic could also affect the amount of overlap a social group has in the places they frequent. Given the significant role of socially structured human mobility in determining fine-scale DENV transmission rates [23], accounting for the dynamic nature of social contacts during a symptomatic DENV infection could allow for more accurate modeling of disease transmission and the design of more efficient disease prevention strategies.

## Supporting information

S1 Text Participant description. Gives participant demographics and data on the number of movement surveys collected at each time point.
(DOCX)

S1 Table Number of participants with data on each day post-symptom onset. In the bottom section, the number of participants for each 3-day group is given.
(PDF)

S2 Table Results of pairwise Wilcoxon Sign Rank tests of paired data for time points pre-, during, and post-illness. Tests were performed for number of locations visited, number of houses visited, and proportion of time spent at home, comparing between three time points: pre-, during, and post-illness. (* p<0.05, ** p<0.01, ***p<0.001).
(PDF)

S3 Table Results of McNemar’s χ^2^ test for time points pre-, during, and post-illness. Tests were performed for number of locations visited, number of houses visited, and proportion of time spent at home, comparing between three time points: pre-,during, and post-illness. (* p<0.05, ** p<0.01, ***p<0.001).
(PDF)

S4 Table Mean predicted probability of a specific location type being visited throughout illness. Probabilities are predicted for pre-, during, and post-illness time periods, based on logistic GLMMs. For houses, the probabilities are predicted for visiting family member’s houses (vs friend’s houses) (Table 1).
(PDF)

S5 Table Results of pairwise Wilcoxon Sign Rank tests of paired data for time points during illness. Tests were performed for number of locations visited, number of houses visited, and proportion of time spent at home, comparing between post-illness and three time points during illness (days 1–3, 4–6, 7–9). (* p<0.05, ** p<0.01, ***p<0.001).
(PDF)

S6 Table Results of McNemar’s χ^2^ test for time points during illness. Tests were performed for number of locations visited, number of houses visited, and proportion of time spent at home, comparing between four time points: days 1–3, 4–6, 7–9, and post-illness. (* p<0.05, ** p<0.01, ***p<0.001).
(PDF)

S7 Table Mean predicted probability of a specific location type being visited during illness. Probabilities are predicted for time points during illness and post-illness, based on logistic GLMMs. For houses, the probabilities are predicted for visiting family member’s houses (vs friend’s houses) (Table 2).
(PDF)

S8 Table Results from likelihood ratio tests between pairs of GLMMs of total number of locations visited with various explanatory variables. The Chi square test statistic is looking at the reduction in deviance for each model as compared to GLMM(day). AICs are also provided for each model. The best-fit model is highlighted in red.
(PDF)

S9 Table Table comparing additive regression models for total number of locations visited with various explanatory variables. AIC values, degrees of freedom (DF), and amount of deviance explained (%) are provided for each model. The best-fit model is highlighted in red.
(PDF)

S10 Table Fixed effects of the best-fit model for total number of locations visited: GLMM “Total Locations ~ day”. (PDF)

S11 Table Results from likelihood ratio tests between pairs of GLMMs of total number of houses visited with various explanatory variables. The Chi square test statistic is looking at the reduction in deviance for each model as compared to GLMM(day). AICs are also provided for each model. The best-fit model is highlighted in red.
(PDF)

S12 Table Table comparing additive regression models for total number of houses visited with various explanatory variables. AIC values, degrees of freedom (DF), and amount of deviance explained (%) are provided for each model. The best-fit model is highlighted in red.
(PDF)

S13 Table Fixed effects of the best-fit model for total number of houses visited: GLMM “Total Houses ~ day”. (PDF)

S1 Fig Average number of houses visited throughout illness. Expressed as the average number of houses visited during each time period. Significant differences, denoted by letter, were found using pairwise paired t-tests with Holm’s correction to account for a family-wise error-rate of 0.05.
(TIF)

S2 Fig Average number of houses visited during illness (in 3-day intervals). Expressed as the average number of locations visited per 3-day period for time point. Significant differences, denoted by letter, were found using pairwise paired t-tests with Holm’s correction to account for a family-wise error-rate of 0.05.
(TIFF)

S3 Fig Median (interquartile range) QWB-score as a function of day of illness. Numbers on top indicate number of surveys that included paired movement and QWB data.
(TIFF)

S1 File Daily Retrospective Movement Survey (DRMS). Lists all survey questions and all possible answers for multiple-choice questions.
(PDF)

S2 File XML code for Daily Retrospective Movement Survey (DRMS). XML code to recreate DRMS survey.
(TXT)

S1 Checklist STROBE Checklist. Checklist used for reporting observational studies.
(DOC)
