# Correction: Dengue illness impacts daily human mobility patterns in Iquitos, Peru

**DOI:** 10.1371/journal.pntd.0008348

**Published:** 2020-06-01

**Authors:** Kathryn L. Schaber, Valerie A. Paz-Soldan, Amy C. Morrison, William H. D. Elson, Alan L. Rothman, Christopher N. Mores, Helvio Astete-Vega, Thomas W. Scott, Lance A. Waller, Uriel Kitron, John P. Elder, Christopher M. Barker, T. Alex Perkins, Gonzalo M. Vazquez-Prokopec

In the published version of this article [1], the Y-axis is labelled incorrectly in Fig 3B. A corrected version of the figure is provided here.

**Fig 3:**
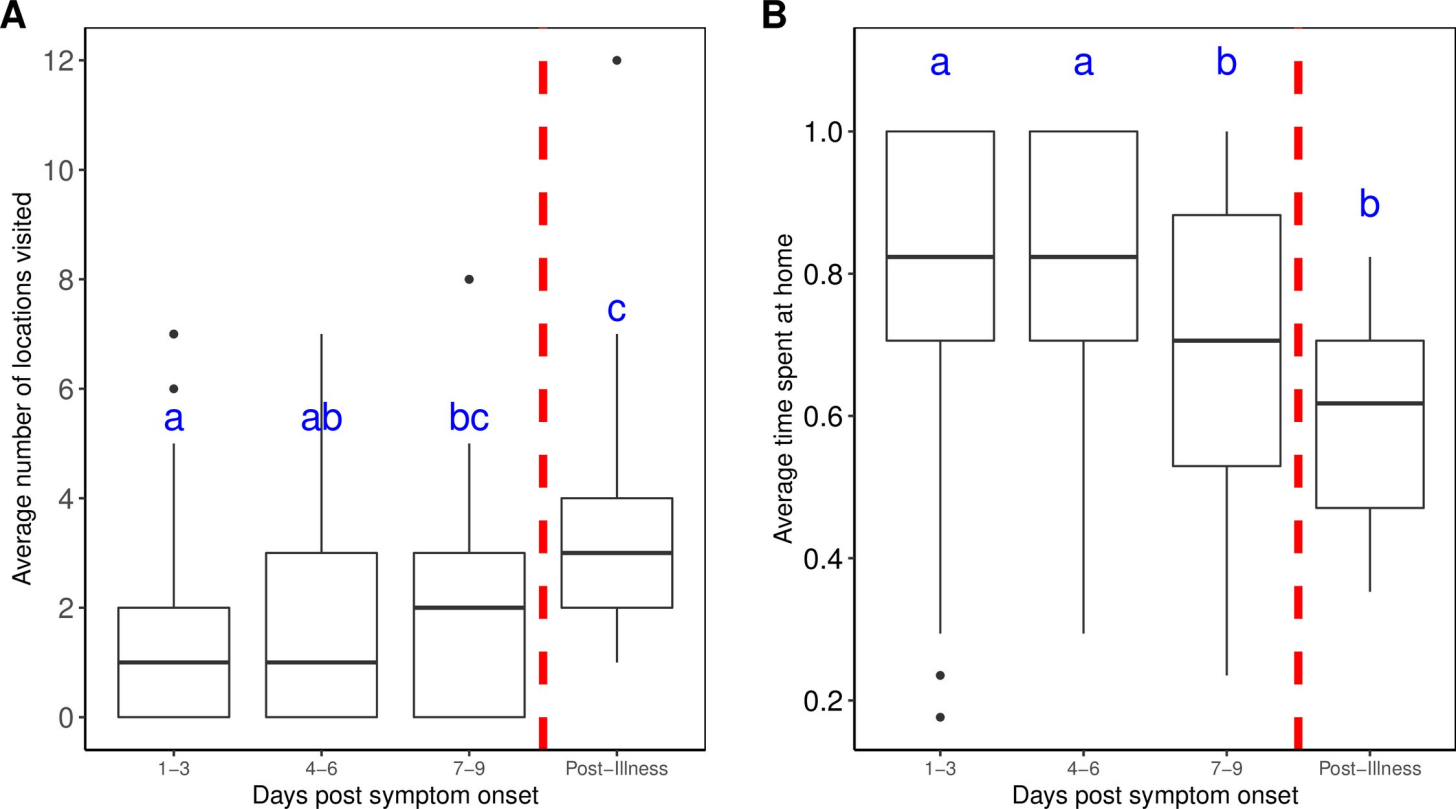
Mobility values during illness (in 3-day intervals). (A) Average number of locations visited per 3-day period. (B) Average proportion of time spent at home per 3-day period. Significant differences, denoted by letters, were detected using pairwise paired Wilcoxon Sign Rank tests with Bonferroni’s correction to account for a family-wise error-rate of 0.05. All significant differences had p-values < 0.05.

The authors apologize for the error in the published article.
