# Supplementary material for: Dengue illness impacts daily human mobility patterns in Iquitos, Peru
Source: PLoS Negl Trop Dis. 2019 Sep 23;13(9):e0007756. doi: 10.1371/journal.pntd.0007756 (PMC6776364; doi:10.1371/journal.pntd.0007756)
Supplement: S1 Text — Gives participant demographics and data on the number of movement surveys collected at each time point. (DOCX) [file pntd.0007756.s001.docx]

**S1 Text**

**Participant Description**

Detailed mobility data were collected from a total of 62 DENV+ participants. The median age of participants was 17 years old, with 35 (57%) participants being under 18 years old. Thirty-nine (63%) participants were students. Other participants were housewives (13%) or worked in unskilled labor (10%), in construction (6%), as vendors (5%), in healthcare (2%), or as self-employed (2%). Of the 62 participants, 35 (60%) were male and 27 (40%) were female. DENV positive participants were administered the pre-illness RMS, a median of 3 days after symptom onset. Of these 62 participants, 34 completed a post-illness mobility survey a range of 30-127 days after initial PCR+ blood test. Daily mobility data was collected from an average of 40 participants on days 1-9 after symptom onset; however, on days 1, 2, and 9, only 21, 33, and 28 participants, respectively, provided data (Table S1). By merging DRMS results into 3-day groups, we had data for 46, 54, and 49 participants on days 1-3, 4-6, and 7-9 after symptom onset, respectively.
