# Supplementary material for: Dengue illness impacts daily human mobility patterns in Iquitos, Peru
Source: PLoS Negl Trop Dis. 2019 Sep 23;13(9):e0007756. doi: 10.1371/journal.pntd.0007756 (PMC6776364; doi:10.1371/journal.pntd.0007756)
Supplement: S1 Table — In the bottom section, the number of participants for each 3-day group is given. (PDF) [file pntd.0007756.s002.pdf]

S1 Table. Number of participants with data on each day post-symptom onset. In the bottom section, the number of participants for each 3-day group is given.

Day post symptom onset	1	2	3	4	5	6	7	8	9	Post-illness
Number of participants with data	21	33	45	51	48	47	45	45	28	34

Day post symptom onset	1-3	4-6	7-9	Post-illness
Number of participants with data	46	54	49	34
