# Supplementary material for: Dengue illness impacts daily human mobility patterns in Iquitos, Peru
Source: PLoS Negl Trop Dis. 2019 Sep 23;13(9):e0007756. doi: 10.1371/journal.pntd.0007756 (PMC6776364; doi:10.1371/journal.pntd.0007756)
Supplement: S2 Table — Tests were performed for number of locations visited, number of houses visited, and proportion of time spent at home, comparing between three time points: pre-, during, and post-illness. (* p<0.05, ** p<0.01, ***p<0.001). (PDF) [file pntd.0007756.s003.pdf]

S2 Table. Results of pairwise Wilcoxon Sign Rank tests of paired data for time points pre-, during, and post-illness. Tests were performed for number of locations visited, number of houses visited, and proportion of time spent at home, comparing between three time points: pre-, during, and post-illness. (* p<0.05, ** p<0.01, ***p<0.001).

Outcome Variable	Time point 1	Time point 2	p-value
Locations visited	During Illness (Days 1-9)	Pre-illness	< 0.001 ***
Locations visited	During Illness (Days 1-9)	Post-illness	0.010 *
Locations visited	Pre-illness	Post-illness	1.000
Houses visited	During Illness (Days 1-9)	Pre-illness	< 0.001 ***
Houses visited	During Illness (Days 1-9)	Post-illness	0.093
Houses visited	Pre-illness	Post-illness	1.000
Time spent at home	During Illness (Days 1-9)	Pre-illness	< 0.001 ***
Time spent at home	During Illness (Days 1-9)	Post-illness	< 0.001 ***
Time spent at home	Pre-illness	Post-illness	1.000
