# Supplementary material for: Dengue illness impacts daily human mobility patterns in Iquitos, Peru
Source: PLoS Negl Trop Dis. 2019 Sep 23;13(9):e0007756. doi: 10.1371/journal.pntd.0007756 (PMC6776364; doi:10.1371/journal.pntd.0007756)
Supplement: S3 Table — Tests were performed for number of locations visited, number of houses visited, and proportion of time spent at home, comparing between three time points: pre-,during, and post-illness. (* p<0.05, ** p<0.01, ***p<0.001). (PDF) [file pntd.0007756.s004.pdf]

S3 Table. Results of McNemar's χ^2 test for time points pre-, during, and post-illness. Tests were performed for number of locations visited, number of houses visited, and proportion of time spent at home, comparing between three time points: pre-,during, and post-illness. (* $p<0.05$, ** $p<0.01$, ***$p<0.001$).

Outcome Variable	Time point 1	Time point 2	χ^2 score	p-value
Locations visited	During Illness (Days 1-9)	Pre-illness	5.82	0.016 *
Locations visited	During Illness (Days 1-9)	Post-illness	3.2	0.074
Locations visited	Pre-illness	Post-illness	0.5	0.480
Houses visited	During Illness (Days 1-9)	Pre-illness	20.35	< 0.001 ***
Houses visited	During Illness (Days 1-9)	Post-illness	4.08	0.043 *
Houses visited	Pre-illness	Post-illness	0.1	0.752
Time spent at home	During Illness (Days 1-9)	Pre-illness	5.82	0.016 *
Time spent at home	During Illness (Days 1-9)	Post-illness	3.2	0.074
Time spent at home	Pre-illness	Post-illness	0	1
