# Supplementary material for: Dengue illness impacts daily human mobility patterns in Iquitos, Peru
Source: PLoS Negl Trop Dis. 2019 Sep 23;13(9):e0007756. doi: 10.1371/journal.pntd.0007756 (PMC6776364; doi:10.1371/journal.pntd.0007756)
Supplement: S4 Table — Probabilities are predicted for pre-, during, and post-illness time periods, based on logistic GLMMs. For houses, the probabilities are predicted for visiting family member’s houses (vs friend’s houses) (Table 1). (PDF) [file pntd.0007756.s005.pdf]

S4 Table. Mean predicted probability of a specific location type being visited throughout illness. Probabilities are predicted for pre-, during, and post-illness time periods, based on logistic GLMMs. For houses, the probabilities are predicted for visiting family member's houses (vs friend's houses) (Table 1).

	Pre-illness (Day 0)	Post-illness (Day 30)	During Illness (Daily)
Education/Work	0.345	0.517	0.243
Health	0.017	0.001	0.316
Other	0.326	0.273	0.255
House	0.304	0.207	0.178
Family's House	0.55	0.46	0.67
