# Supplementary material for: Dengue illness impacts daily human mobility patterns in Iquitos, Peru
Source: PLoS Negl Trop Dis. 2019 Sep 23;13(9):e0007756. doi: 10.1371/journal.pntd.0007756 (PMC6776364; doi:10.1371/journal.pntd.0007756)
Supplement: S5 Table — Tests were performed for number of locations visited, number of houses visited, and proportion of time spent at home, comparing between post-illness and three time points during illness (days 1–3, 4–6, 7–9). (* p<0.05, ** p<0.01, ***p<0.001). (PDF) [file pntd.0007756.s006.pdf]

S5 Table. Results of pairwise Wilcoxon Sign Rank tests of paired data for time points during illness. Tests were performed for number of locations visited, number of houses visited, and proportion of time spent at home, comparing between post-illness and three time points during illness (days 1-3, 4-6, 7-9). (* p<0.05, ** p<0.01, ***p<0.001).

Outcome Variable	Time point 1	Time point 2	p-value
Locations visited	Days 1-3	Post-illness	0.0170 *
Locations visited	Days 4-6	Post-illness	< 0.001 ***
Locations visited	Days 7-9	Post-illness	0.123
Locations visited	Days 1-3	Days 4-6	1.000
Locations visited	Days 1-3	Days 7-9	0.047 *
Locations visited	Days 4-6	Days 7-9	0.871
Houses visited	Days 1-3	Post-illness	0.628
Houses visited	Days 4-6	Post-illness	0.148
Houses visited	Days 7-9	Post-illness	0.722
Houses visited	Days 1-3	Days 4-6	1.000
Houses visited	Days 1-3	Days 7-9	0.289
Houses visited	Days 4-6	Days 7-9	1.000
Time spent at home	Days 1-3	Post-illness	0.005 **
Time spent at home	Days 4-6	Post-illness	< 0.001 ***
Time spent at home	Days 7-9	Post-illness	0.308
Time spent at home	Days 1-3	Days 4-6	1.000
Time spent at home	Days 1-3	Days 7-9	0.014 *
Time spent at home	Days 4-6	Days 7-9	0.008 **
