# Supplementary material for: Dengue illness impacts daily human mobility patterns in Iquitos, Peru
Source: PLoS Negl Trop Dis. 2019 Sep 23;13(9):e0007756. doi: 10.1371/journal.pntd.0007756 (PMC6776364; doi:10.1371/journal.pntd.0007756)
Supplement: S6 Table — Tests were performed for number of locations visited, number of houses visited, and proportion of time spent at home, comparing between four time points: days 1–3, 4–6, 7–9, and post-illness. (* p<0.05, ** p<0.01, ***p<0.001). (PDF) [file pntd.0007756.s007.pdf]

S6 Table. Results of McNemar's χ^2 test for time points during illness. Tests were performed for number of locations visited, number of houses visited, and proportion of time spent at home, comparing between four time points: days 1-3, 4-6, 7-9, and post-illness. (* $p<0.05$, ** $p<0.01$, ***$p<0.001$).

Outcome Variable	Time point 1	Time point 2	χ^2 score	p-value
Locations visited	Days 1-3	Post-illness	10.08	0.001 **
Locations visited	Days 4-6	Post-illness	11.08	< 0.001 ***
Locations visited	Days 7-9	Post-illness	7.11	0.008 **
Locations visited	Days 1-3	Days 4-6	0	1.000
Locations visited	Days 1-3	Days 7-9	3.06	0.080
Locations visited	Days 4-6	Days 7-9	2.08	0.149
Houses visited	Days 1-3	Post-illness	2.29	0.131
Houses visited	Days 4-6	Post-illness	2.5	0.114
Houses visited	Days 7-9	Post-illness	0.44	0.505
Houses visited	Days 1-3	Days 4-6	0	1.000
Houses visited	Days 1-3	Days 7-9	5.14	0.023 *
Houses visited	Days 4-6	Days 7-9	3.2	0.074
Time spent at home	Days 1-3	Post-illness	7.11	0.008 **
Time spent at home	Days 4-6	Post-illness	7.11	0.008 **
Time spent at home	Days 7-9	Post-illness	3.2	0.074
Time spent at home	Days 1-3	Days 4-6	0	1.000
Time spent at home	Days 1-3	Days 7-9	4	0.046 *
Time spent at home	Days 4-6	Days 7-9	2.5	0.114
