# Supplementary material for: Dengue illness impacts daily human mobility patterns in Iquitos, Peru
Source: PLoS Negl Trop Dis. 2019 Sep 23;13(9):e0007756. doi: 10.1371/journal.pntd.0007756 (PMC6776364; doi:10.1371/journal.pntd.0007756)
Supplement: S7 Table — Probabilities are predicted for time points during illness and post-illness, based on logistic GLMMs. For houses, the probabilities are predicted for visiting family member’s houses (vs friend’s houses) (Table 2). (PDF) [file pntd.0007756.s008.pdf]

S7 Table. Mean predicted probability of a specific location type being visited during illness. Probabilities are predicted for time points during illness and post-illness, based on logistic GLMMs. For houses, the probabilities are predicted for visiting family member’s houses (vs friend’s houses) (Table 2).

	Days 1-3	Days 4-6	Days 7-9	Post-Illness (Day 30)
Education/Work	0.165	0.244	0.278	0.479
Health	0.467	0.353	0.177	0.002
Other	0.200	0.208	0.323	0.315
House	0.138	0.175	0.193	0.200
Family’s House	0.71	0.67	0.65	0.46
