# Supplementary material for: Dengue illness impacts daily human mobility patterns in Iquitos, Peru
Source: PLoS Negl Trop Dis. 2019 Sep 23;13(9):e0007756. doi: 10.1371/journal.pntd.0007756 (PMC6776364; doi:10.1371/journal.pntd.0007756)
Supplement: S8 Table — The Chi square test statistic is looking at the reduction in deviance for each model as compared to GLMM(day). AICs are also provided for each model. The best-fit model is highlighted in red. (PDF) [file pntd.0007756.s009.pdf]

S8 Table. Results from likelihood ratio tests between pairs of GLMMs of total number of locations visited with various explanatory variables. The Chi square test statistic is looking at the reduction in deviance for each model as compared to GLMM(day). AICs are also provided for each model. The best-fit model is highlighted in red.

MODEL	DF	AIC	Deviance	Chisq	Pr(>Chi)
GLMM(day)	3	368	362.25		
GLMM(qwb_score)	3	372	365.72	0	1
GLMM(day, qwb_score)	4	369	360.71	1.55	0.21
GLMM(day*qwb_score)	5	370	360.45	1.81	0.41
