# Supplementary material for: Dengue illness impacts daily human mobility patterns in Iquitos, Peru
Source: PLoS Negl Trop Dis. 2019 Sep 23;13(9):e0007756. doi: 10.1371/journal.pntd.0007756 (PMC6776364; doi:10.1371/journal.pntd.0007756)
Supplement: S10 Table — (PDF) [file pntd.0007756.s011.pdf]

S10 Table. Fixed effects of the best-fit model for total number of locations visited: GLMM "Total Locations ~ day".

	Estimate	Std. Error	t value	p-value
Intercept	-0.898	0.274	-3.28	0.001 **
day	0.077	0.041	1.86	0.063
