# Supplementary material for: Dengue illness impacts daily human mobility patterns in Iquitos, Peru
Source: PLoS Negl Trop Dis. 2019 Sep 23;13(9):e0007756. doi: 10.1371/journal.pntd.0007756 (PMC6776364; doi:10.1371/journal.pntd.0007756)
Supplement: S11 Table — The Chi square test statistic is looking at the reduction in deviance for each model as compared to GLMM(day). AICs are also provided for each model. The best-fit model is highlighted in red. (PDF) [file pntd.0007756.s012.pdf]

S11 Table. Results from likelihood ratio tests between pairs of GLMMs of total number of houses visited with various explanatory variables. The Chi square test statistic is looking at the reduction in deviance for each model as compared to GLMM(day). AICs are also provided for each model. The best-fit model is highlighted in red.

MODEL	DF	AIC	Deviance	Chisq	Pr(>Chi)
GLMM(day)	3	103	96.72		
GLMM(qwb_score)	3	104	97.91	0	1
GLMM(day, qwb_score)	4	105	96.59	0.133	0.715
GLMM(day*qwb_score)	5	105	94.65	2.067	0.356
