# Supplementary material for: Dengue illness impacts daily human mobility patterns in Iquitos, Peru
Source: PLoS Negl Trop Dis. 2019 Sep 23;13(9):e0007756. doi: 10.1371/journal.pntd.0007756 (PMC6776364; doi:10.1371/journal.pntd.0007756)
Supplement: S12 Table — AIC values, degrees of freedom (DF), and amount of deviance explained (%) are provided for each model. The best-fit model is highlighted in red. (PDF) [file pntd.0007756.s013.pdf]

S12 Table. Table comparing additive regression models for total number of houses visited with various explanatory variables. AIC values, degrees of freedom (DF), and amount of deviance explained (%) are provided for each model. The best-fit model is highlighted in red.

MODEL	DF	AIC	Deviance Explained (%)
MODEL	DF	AIC	Deviance Explained (%)
GAMM(day)	3	841	1.26 %
GAMM(te(qwb_score,day))	7	853	2.52%
