# Supplementary material for: Dengue illness impacts daily human mobility patterns in Iquitos, Peru
Source: PLoS Negl Trop Dis. 2019 Sep 23;13(9):e0007756. doi: 10.1371/journal.pntd.0007756 (PMC6776364; doi:10.1371/journal.pntd.0007756)
Supplement: S13 Table — (PDF) [file pntd.0007756.s014.pdf]

S13 Table. Fixed effects of the best-fit model for total number of houses visited: GLMM "Total Houses ~ day".

	Estimate	Std. Error	z value	p-value
Intercept	-8.457	2.083	-4.061	< 0.001 ***
day	0.171	0.010	1.718	0.086
