# Supplementary figures and images for: Dengue illness impacts daily human mobility patterns in Iquitos, Peru
Source: PLoS Negl Trop Dis. 2019 Sep 23;13(9):e0007756. doi: 10.1371/journal.pntd.0007756 (PMC6776364; doi:10.1371/journal.pntd.0007756)

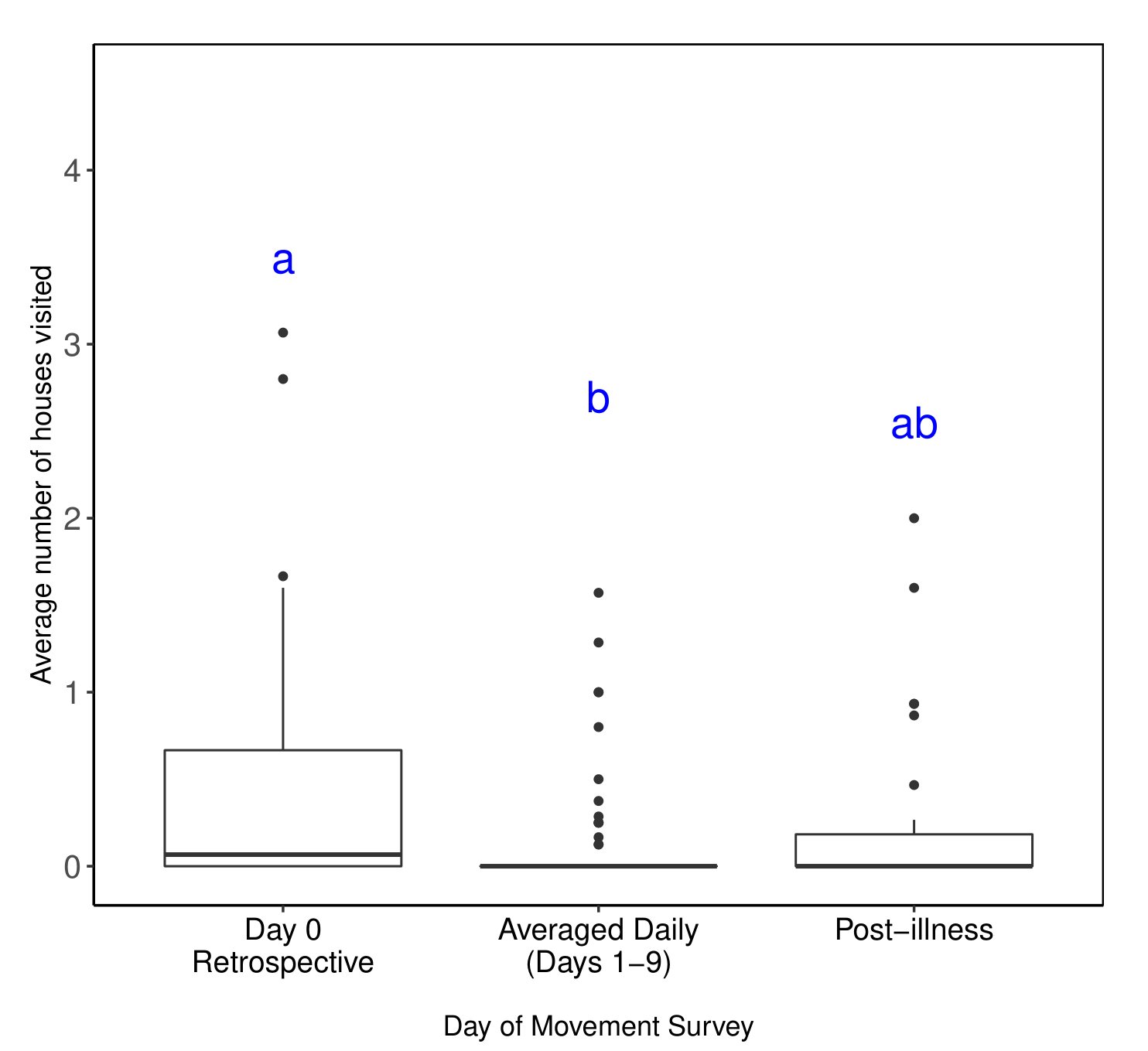

Supplement: S1 Fig — Expressed as the average number of houses visited during each time period. Significant differences, denoted by letter, were found using pairwise paired t-tests with Holm’s correction to account for a family-wise error-rate of 0.05. (TIF) [file pntd.0007756.s015.tif]

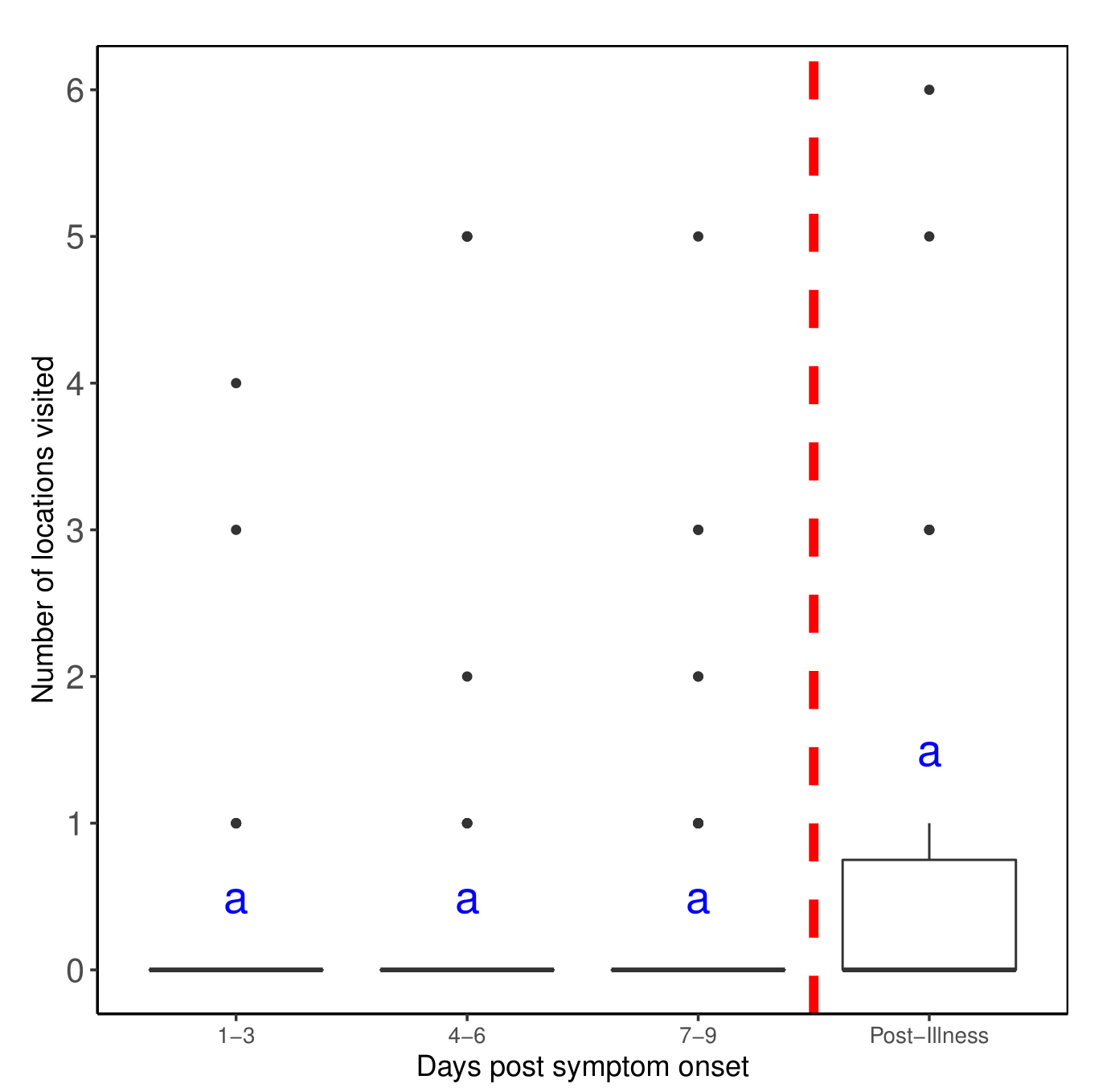

Supplement: S2 Fig — Expressed as the average number of locations visited per 3-day period for time point. Significant differences, denoted by letter, were found using pairwise paired t-tests with Holm’s correction to account for a family-wise error-rate of 0.05. (TIFF) [file pntd.0007756.s016.tiff]

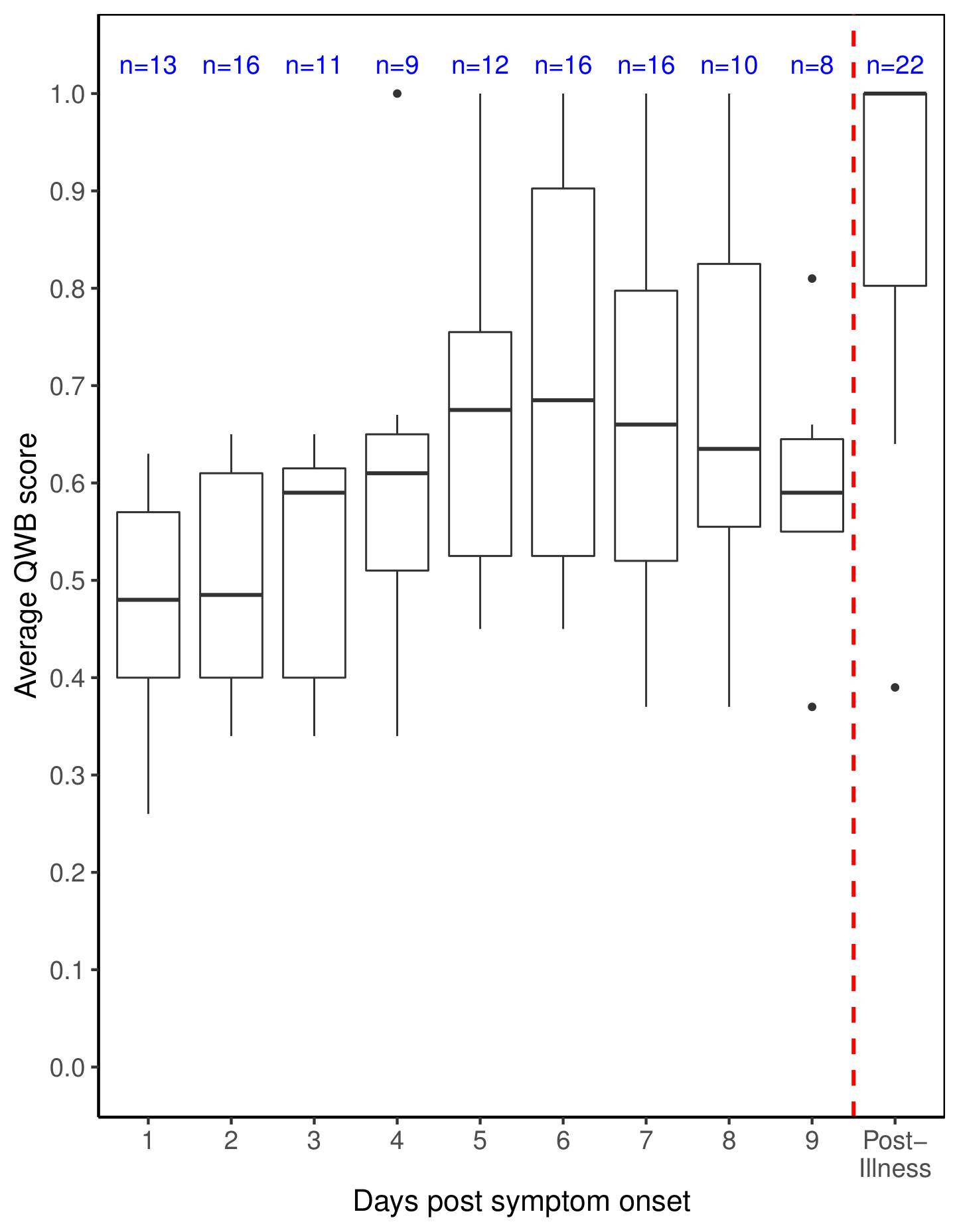

Supplement: S3 Fig — Numbers on top indicate number of surveys that included paired movement and QWB data. (TIFF) [file pntd.0007756.s017.tiff]
