# Supplementary material for: Dengue illness impacts daily human mobility patterns in Iquitos, Peru
Source: PLoS Negl Trop Dis. 2019 Sep 23;13(9):e0007756. doi: 10.1371/journal.pntd.0007756 (PMC6776364; doi:10.1371/journal.pntd.0007756)
Supplement: S1 File — Lists all survey questions and all possible answers for multiple-choice questions. (PDF) [file pntd.0007756.s018.pdf]

Project 2

Retrospective daily (EAR7)

1. El último EAR7 fue realizada en la fecha **____** que corresponde al día **____**
2. Date and time
    1. Actual date of questionnaire required
    2. At what time did you complete the questionnaire?
    3. #form/fecha_hora/actual_date_ear7
3. **____** This date is outside the expected range, is that correct?** required
    1. Yes
4. Interview details
    1. The interview is answered by: required
        1. Patient
        2. Parents
        3. Other caregiver
    2. Another caregiver required
    3. How did you conduct the interview? required
        1. By phone
        2. In person
5. Interviews: How often? required
    1. Daily
    2. Spending a day
    3. Spending two days
    4. Passing 3 days
6.
    1. What is the cluster number? required
    2. What was the diagnosis? required
        1. Zika
        2. Dengue 1
        3. Dengue 2
        4. Dengue 3
        5. Dengue 4
        6. Chikungunya
    3. ¿Esta persona es el caso indice o contacto? required
        1. Index
        2. Contact
    4. #form/diagnosis_details/cluster_code
    5. #form/diagnosis_details/message_day30
7. GPS
    1. Accepts GPS? required
        1. Yes
        2. No
    2. GPS code required
8. Please check the temperature
    1. Which method did you use to take the temperature? required
        1. Tympanic
        2. Axillary
        3. I didn't take it
    2. Temperature right: required
    3. Temperature left: required
9. Section: Place of work or study daily
10. Places of work or study daily
    1. ¿Workplace or study mentioned earlier?
        1. No
        2. Yes
    2. Place of work or study daily
        1. Number of work / study in the initial retrospective
        2. Work or study?
            1. Work
            2. Estudio
        3. Code of the place of work or study daily
        4. Name of place
        5. Street name
        6. Street number
        7. Reference place
        8. District
            1. Belem
            2. Iquitos
            3. Punchana
            4. San Juan Bautista

3. Day they went to work or study
    1. Day they went to work or study
        1. Day required
            1. Day 0
            2. Day 1
            3. Day 2
            4. Day 3
            5. Day 4
            6. Day 5
            7. Day 6
            8. Day 7
    2. You have selected day 0, which corresponds to **____**. Please check that this is the correct date.
    3. You have selected day 1, which corresponds to **____**. Please check that this is the correct date.
    4. You have selected day 2, which corresponds to **____**. Please check that this is the correct date.
    5. You have selected day 3, which corresponds to **____**. Please check that this is the correct date.
    6. You have selected day 4, which corresponds to **____**. Please check that this is the correct date.
    7. You have selected day 5, which corresponds to **____**. Please check that this is the correct date.
    8. You have selected day 6, which corresponds to **____**. Please check that this is the correct date.
    9. You have selected day 7, which corresponds to **____**. Please check that this is the correct date.
    10. Day was to work or study
        1. How many times did you visit in the last two weeks? required
        2. At what time of day was it? required
            1. Morning
            2. Afternoon
            3. Night
        3. How much time did you spend? required
            1. 15 - 30 minutes
            2. 30 minutes - 1hour
            3. 1 - 2 hours
            4. 2 - 4 hours
            5. 4 - 6 hours
            6. 6 - 8 hours
            7. more than 8 hours
        4. He revisited this place
4. Did you visit other work / study?

11. Section: Houses visited daily
12. Houses visited daily
    1. House visited
        1. Had you visited this house before?
            1. No
            2. Yes
    2. House visited
        1. House number in the initial retrospective survey
        2. Code of house visited daily
        3. Visited house of:
            1. Grandparent's house
            2. Parent's house
            3. Brother's house
            4. Child's house
            5. Uncle/aunt's house
            6. Cousin's house
            7. Nephew's house
            8. Boyfriend/girlfriend's house
            9. Friend's house
            10. father in law house
            11. brother in law house
            12. Someone else's home
        4. Nombre de persona que visitó
        5. Street
        6. House number
        7. Reference house
        8. District
            1. Belem
            2. Iquitos
            3. Punchana
            4. San Juan Bautista
        9. Do you visit this place at least 1 time every two weeks?
            1. No
            2. Yes
    3. Day visit note
    4. Day they visited the house
        1. Day they visited the house
            1. Day the visited the house required
                1. Day 0
                2. Day 1
                3. Day 2
                4. Day 3
                5. Day 4
                6. Day 5
                7. Day 6
                8. Day 7

2. You have selected day 0, which corresponds to **____**. Please check that this is the correct date.
3. You have selected day 1, which corresponds to **____**. Please check that this is the correct date.
4. You have selected day 2, which corresponds to **____**. Please check that this is the correct date.
5. You have selected day 3, which corresponds to **____**. Please check that this is the correct date.
6. You have selected day 4, which corresponds to **____**. Please check that this is the correct date.
7. You have selected day 5, which corresponds to **____**. Please check that this is the correct date.
8. You have selected day 6, which corresponds to **____**. Please check that this is the correct date.
9. You have selected day 7, which corresponds to **____**. Please check that this is the correct date.
10. Day they visited the house
    1. How many times it was from the last time you interviewed him? required
    2. How much time did you spend in this location? required
        1. 15 - 30 minutes
        2. 30 minutes - 1hour
        3. 1 - 2 hours
        4. 2 - 4 hours
        5. 4 - 6 hours
        6. 6 - 8 hours
        7. more than 8 hours
    3. At what time of day was it? required
        1. Morning
        2. Afternoon
        3. Night
    4. Reason for visiting this house
        1. Informational support
        2. Resources/Logistics
        3. Emotional support
        4. Unrelated to disease
        5. Another reason to visit
    5. He revisited the house

5. Was there any change in the time you usually visit this house?
    1. No
    2. Yes
    3. I do not remember
6. He changed the time visiting this house
    1. Why was there a change?
        1. Disease
        2. Another reason for change visit
    2. What is the other reason?
    3. Has anyone in this house been ill in the last 15 days?
        1. No
        2. Yes
        3. None known
    4. Sketch house?
        1. No
        2. Yes
7. Sketch of house visited
8. Did you visit another house?

13. Section: visitors recieved
14. People who visited me daily
    1. Who visited me
        1. Visitor's name
        2. Relationship with the visitor
            1. Family
            2. Friend
            3. Neighbor
            4. Another relationship
    2. Section: visitors recieved
    3. Day they visited me (the visitor)
        1. Day they visited me (the visitor)
            1. Day they visited me (the visitor) required
                1. Day 0
                2. Day 1
                3. Day 2
                4. Day 3
                5. Day 4
                6. Day 5
                7. Day 6
                8. Day 7
        2. You have selected day 0, which corresponds to **____**. Please check that this is the correct date.
        3. You have selected day 1, which corresponds to **____**. Please check that this is the correct date.
        4. You have selected day 2, which corresponds to **____ **. Please check that this is the correct date.
        5. You have selected day 3, which corresponds to **____**. Please check that this is the correct date.
        6. You have selected day 4, which corresponds to **____**. Please check that this is the correct date.
        7. You have selected day 5, which corresponds to **____**. Please check that this is the correct date.
        8. You have selected day 6, which corresponds to **____**. Please check that this is the correct date.
        9. You have selected day 7, which corresponds to **____**. Please check that this is the correct date.

10. Visited daily details
    1. Does this person visited directly to you?
        1. No
        2. Yes
    2. Did this person know of your illness?
        1. No
        2. Yes
        3. None known
    3. Reason why this person visited me
        1. Informational support
        2. Resources/Logistics
        3. Emotional support
        4. Unrelated to disease
        5. Another reason to visit
11. He revisited this person
4. Does this person visit you at least once a week?
    1. No
    2. Yes
5. Another person visited him?

15. Section: Places visited daily
16. Places visited daily
    1. Type of place
        1. Market
        2. Ports
        3. Recreation
        4. Education
        5. Restaurante
        6. Health
        7. Church
        8. Cemetery
        9. Internet
        10. Elsewhere
    2. market
        1. Belen market
        2. Central market
        3. Productores market
        4. Modelo market
        5. Clavero market
        6. Nanay market
        7. Urarinas market
        8. Other market
        9. La Nortenhita
    3. Ports
        1. Fishing port
        2. Masusa port
        3. Belen port
        4. Henrry port
        5. Other port
    4. Recreation
        1. Boulevard
        2. Plaza de armas
        3. Plaza Estela Maris
        4. Plaza San Antonio
        5. Plaza Grau
        6. Plaza July 28
        7. Zonal Park
        8. Max Augustin Stadium
        9. CNI Complex
        10. Consortium priests
        11. C. C. Pardo
        12. Multiplexes Iquitos
        13. Other Recreation
    5. Education
        1. Barcia Bonifati Garden
        2. Tupac School
        3. Clavero School
        4. Claverito School
        5. Sagrado Corazon School
        6. Maynas School
        7. Inmaculada School
        8. Unap
        9. UCP
        10. UPO
        11. Other Education

6. Restaurant
    1. Restaurant Kikiriki
    2. Restaurant Rico Rico
    3. Restaurant Trinchero
    4. Chifa Li Chinito
    5. Restaurant Sabroso Pollo
    6. Chifa Hua Xing
    7. Other Restaurant
7. Health
    1. Regional hospital
    2. Iquitos hospital
    3. Essalud
    4. Health Post
    5. Health Center
    6. Clinic
8. Church
    1. Church Matriz
    2. church_Church Inmaculada
    3. Church Fatima
    4. Church Amazonas
    5. Church Salud
    6. Church San Martin
    7. Other_church
9. Cemetery
    1. General cemetery
    2. Cemetery punchana
    3. Cematary angels
    4. Gardens of Eden
    5. Other Cemetery
10. Internet
    1. Other internet
11. Place others
    1. Sedaloreto
    2. Electro oriente
    3. Telefonica
    4. National bank
    5. Quistococha
    6. Caja Maynas
    7. Other Elsewhere
12. Which UNAP location?
    1. Zungarococha
    2. Bermudez/Bolognesi(Aulas)
    3. Pevas/Samanez Ocampo(FCACENIT)
    4. Pevas/Samanez Ocampo(BIBLIOTECA)
    5. Pevas Cuadra 5(AULA MAGNA)
    6. Pevas/Nanay(BIOLOGÍA)
    7. Nauta/Samanez Ocampo(AGRONOMÍA)
    8. Sargento Lores cuadra 6 (Educación
    9. Nanay cuadra 3(COMEDOR)
    10. Nanay cuadra 3(FCACENIT)
    11. San Lorenzo/ Av. La Marina(SIRNA)
    12. (ODONTOLOGÍA)
    13. Av. Circumvalación/Colonial(MEDICINA)
    14. Dina Limaco(ENFERMERIA)
    15. Los rosales (Post Grado)
13. Which Kikiriki restaurant?
    1. Condamine/napo
    2. Av. Quiñones/Los Rosales
    3. Alfonso Ugarte/Grau
    4. Freyre con San Jose
14. Which Rico rico restaurant?
    1. Av. Quiñones/Miraflores
    2. Nauta/Fitzcarrald
15. Which Trinchero restaurant?
    1. Condamine/napo
    2. Av. Quiñonez/Guardia Civil
16. Which Essalud location?
    1. Av. La Marina Km 2.5
    2. Av. Del Ejército/Garisho
    3. Trujillo/Diego de Almagro
    4. Av. Quiñones Km 3
17. Which health post?
    1. Health post name
    2. Health post address
18. Which health center?
    1. Health center name
    2. Health center address
19. Which health clinic?
    1. Health clinic name
    2. Health clinic address

20. Which Sedaloreto location?
    1. Guardia Civil/19 de Julio
    2. Huallaga cuadra 3
21. Which Electro Oriente location?
    1. Av. Freyre/San Antonio
    2. Tacna/Putumayo
    3. Arica cuadra 7
22. Which Telefonica location?
    1. Arica Cuadra 2 (central)
    2. Arica Cuadra 2 (pago)
    3. Huallaga/napo
23. Which Banco de la Nacion location?
    1. Condamine/Yavari
    2. Av. 28 de Julio/Amazonas
    3. Nanay/Loreto
    4. Av. Quiñones/Los Rosales
24. Which Caja maynas location?
    1. Prospero/Ucayali
    2. Av. 28 de Julio/
    3. Arequipa cuadra 4
    4. Av. Quiñones Km 3
25. Which other place?
    1. Place name
    2. Place address
26. Día que visitó otro lugar
    1. Day in this location
        1. Day in this location required
            1. Day 0
            2. Day 1
            3. Day 2
            4. Day 3
            5. Day 4
            6. Day 5
            7. Day 6
            8. Day 7
    2. You have selected day 0, which corresponds to **____**. Please check that this is the correct date.
    3. You have selected day 1, which corresponds to **____**. Please check that this is the correct date.
    4. You have selected day 2, which corresponds to **____ **. Please check that this is the correct date.
    5. You have selected day 3, which corresponds to **____**. Please check that this is the correct date.
    6. You have selected day 4, which corresponds to **____**. Please check that this is the correct date.
    7. You have selected day 5, which corresponds to **____**. Please check that this is the correct date.
    8. You have selected day 6, which corresponds to **____**. Please check that this is the correct date.
    9. You have selected day 7, which corresponds to **____**. Please check that this is the correct date.
    10. Time in this location details
        1. How many times did you visit in the last two weeks? House 1 required
        2. How much time did you spend in this location? required
            1. 15 - 30 minutes
            2. 30 minutes - 1hour
            3. 1 - 2 hours
            4. 2 - 4 hours
            5. 4 - 6 hours
            6. 6 - 8 hours
            7. more than 8 hours
27. Did you visit elsewhere?

17. Section: Daily time in house
18. Day they were at home
    1. Day they were at home
        1. Day they were at home required
            1. Day 0
            2. Day 1
            3. Day 2
            4. Day 3
            5. Day 4
            6. Day 5
            7. Day 6
            8. Day 7
    2. You have selected day 0, which corresponds to **____**. Please check that this is the correct date.
    3. You have selected day 1, which corresponds to **____**. Please check that this is the correct date.
    4. You have selected day 2, which corresponds to **____ **. Please check that this is the correct date.
    5. You have selected day 3, which corresponds to **____**. Please check that this is the correct date.
    6. You have selected day 4, which corresponds to **____**. Please check that this is the correct date.
    7. You have selected day 5, which corresponds to **____**. Please check that this is the correct date.
    8. You have selected day 6, which corresponds to **____**. Please check that this is the correct date.
    9. You have selected day 7, which corresponds to **____**. Please check that this is the correct date.
    10. Hours in the house daily
    11. Add Another time at home?
19. Section: physical state
20. RECORDITORIO! Debe contestar la seccion 'Estado Físico' para cada día, aparte del día 7. Por favor no olvide esto!

21. Physical state
   1. For which day
      1. Day required
         1. Day 0
         2. Day 1
         3. Day 2
         4. Day 3
         5. Day 4
         6. Day 5
         7. Day 6
         8. Día 7
      2. You have selected day 0, which corresponds to **____**. Please check that this is the correct date.
      3. You have selected day 1, which corresponds to **____**. Please check that this is the correct date.
      4. You have selected day 2, which corresponds to **____**. Please check that this is the correct date.
      5. You have selected day 3, which corresponds to **____**. Please check that this is the correct date.
      6. You have selected day 4, which corresponds to **____**. Please check that this is the correct date.
      7. You have selected day 5, which corresponds to **____**. Please check that this is the correct date.
      8. You have selected day 6, which corresponds to **____**. Please check that this is the correct date.
      9. Do not use this form for day 7
   2. How is your ability to complete self-care activities, such as washing, dressing and grooming himself? required
      1. Completely limited
      2. Partially limited
      3. No limits
   3. How is your ability to complete daily activities such as cooking, shopping for food, feed himself life? required
      1. Completely limited
      2. Partially limited
      3. No limits
   4. Are you able to participate in earning a wage or having a role within the household that contributes to supporting yourself and your family? required
      1. No, I completely rely on my family for monetary support.
      2. Yes, I can contribute to the household income but my contribution to the household income is limited.
      3. Yes. I can provide for myself and my family.
22. Monetary contribution to family
23. Observers during visit
   1. Was there any observer?
      1. No
      2. Yes
   2. Relationship to respondent
   3. Was it easy for the participant to complete the survey?
      1. Easy
      2. Más o menos
      3. Difficult
   4. General comment
